# Highly sensitive and accurate detection of cholesterol based on a single red upconversion biosensor†

**DOI:** 10.1039/d3ra07354h

**Published:** 2024-03-06

**Authors:** Pang Tingyuan, Liu Xiaorui, Li Jia, Song Qi, Li Junren, Han Ling, Shu Wenying, Jian Xiaoshun, Zhang Meimei

**Affiliations:** a Department of Pharmacy, Affiliated Cancer Hospital, Institute of Guangzhou Medical University Guangzhou China; b Integrated Traditional Chinese and Western Medicine Department (Internal Medicine Section 5), Affiliated Cancer Hospital & Institute of Guangzhou Medical University Guangzhou China

## Abstract

Cholesterol (CHOL) is an important clinical biochemical indicator that plays an important role in the regulation of the fluidity, permeability, and microstructure of cell membranes. Therefore, it is necessary to accurately monitor CHOL levels in biological samples for the early prevention and diagnosis of various diseases. The single-band red upconversion nanoparticle (UCNP) emits light within the optical transmission window of biological tissues, and can penetrate deeper biological tissues and cause less energy loss due to scattering and thus have higher sensitivity and accuracy. Here, using the nontoxic, sensitive, and photochemically stable 3,3′,5,5′-tetramethylbenzidine (TMB) as the quenching agent and single red UCNP as the fluorescent donor, a dual-readout colorimetric and fluorescent sensor was developed to detect CHOL. The detection mechanism and feasibility were discussed in detail, and experimental conditions such as Fe^2+^ concentration, TMB concentration and reaction time were explored. Under optimal conditions, the limits of CHOL detection by colorimetry and fluorescence were 0.85 μM and 0.63 μM. The sensing system was used to measure CHOL in serum samples and the values obtained by these two modes were close, and the spiked recoveries were 97.2–102.2% and 97.1–103.7%, respectively, which holds great potential in clinical diagnosis and health management.

## Abstract 2

Cholesterol (CHOL) is an important clinical biochemical indicator that plays an important role in the regulation of the fluidity, permeability, and microstructure of cell membranes.

## Graphical abstract

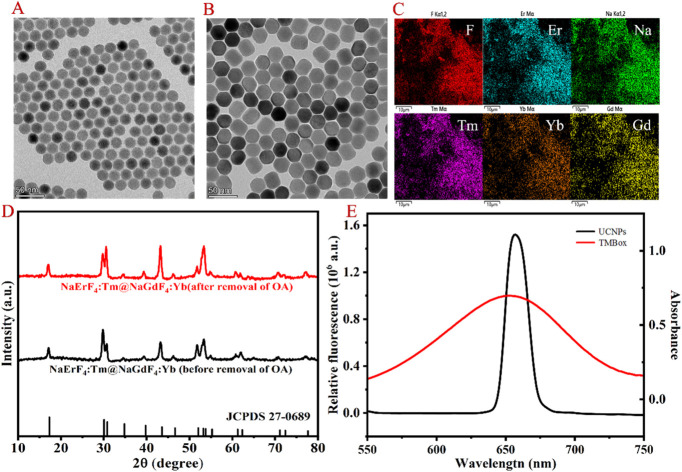

## 1 Introduction

As an essential lipid, cholesterol (CHOL) is the structural component of the hormone system and the only precursor of steroid hormones, and is essential to maintain the integrity of the biofilm and cell structure and the fluidity of cell membranes.^1,2^ Abnormal levels of CHOL in serum are associated with many diseases, for example, excessive levels of CHOL have a connection with the occurrence and development of coronary heart disease, cardiovascular disease, hypertension, and myocardial infarction.^3,4^ Low CHOL levels may also lead to hypolipoproteinemia, sepsis, malnutrition, hyperthyroidism, and liver disease.^5^ Therefore, it is significant to accurately monitor CHOL levels in biological samples for early prevention and diagnosis of related diseases.

In recent years, the reported CHOL detection methods have mainly included electrochemical biosensors,^6^ colorimetry,^7^ chemiluminescence^8^ and fluorescence biosensing.^9^ Among them, the fluorescence method has attracted much attention due to some advantages of sensitivity, stability, and simple operation, but when applied to biological samples, it is limited owing to interference from the spontaneous fluorescence of biological tissues and scattered light. Unlike traditional fluorescent materials, upconversion nanoparticle (UCNP) is inorganic luminescent materials that could transform near-infrared (NIR) excited light into visible or ultraviolet light, with rich energy levels and long-life emission, good photochemical stability, continuous emission capacity, low potential toxicity and relatively narrow bandwidth.^10–12^ Because most substances in the organism do not emit light under the excitation of NIR light, the light scattering caused by biological tissues is greatly decreased and the self-background fluorescence can be ignored. At the same time, the penetration depth of biological tissues becomes greater and can effectively reduce the damage to biological organs and tissues, which makes UCNP capable of being applied to the complex biological internal environment.^13^ More interestingly, red UCNP luminescence falls in the optical transmission window of biological tissue, can penetrate deeper biological tissue, and less energy is lost by scattering, making them ideal sensor luminescent materials. Rakov^14^ prepared red UCNP by doping Er^3+^ and Tm^3+^ in powders of YOF and Y_2_O_3_. However, the synthesized samples had poor morphology and relatively weak red luminescence because of the combustion preparation method. In addition, many researchers directly use KMnF_3_ as a lattice host and uniformly doping Er^3+^ in KMnF_3_ to achieve single-red upconversion emission through energy transfer between Mn^2+^ and Er^3+^ ions. However, the formed cubic phase and the presence of electron vacancies are not conducive to upconversion emission.^15^ In order to overcome the above shortcomings of the preparation of single-band bright red UCNP, Xu^16^ prepared a novel and highly efficient active core–shell UCNP (NaErF_4_ : Tm@NaGdF_4_ : Yb) using Er^3+^ ions as both sensitizer and activator, which emits ultra-bright red by 980 nm excitation. To alleviate the fluorescence quenching induced by energy transfer between Er^3+^ ions and internal lattice defects, a Tm^3+^ capture center is doped in the core to limit the energy of Er^3+^ through energy return (Er^3+^ → Tm^3+^ → Er^3+^). Subsequently, the active shell is grown in the core region, and the high efficiency energy transfer of near infrared photons in the up-conversion region is realized, which inhibits the fluorescence quenching induced by surface defects and surface-related ligands. This research provides a convenient method for preparing single red UCNP, however, the application of biosensors based on red UCNP in disease diagnosis still needs to be developed, especially in the study of CHOL detection in complex biological samples.

**Scheme 1:**
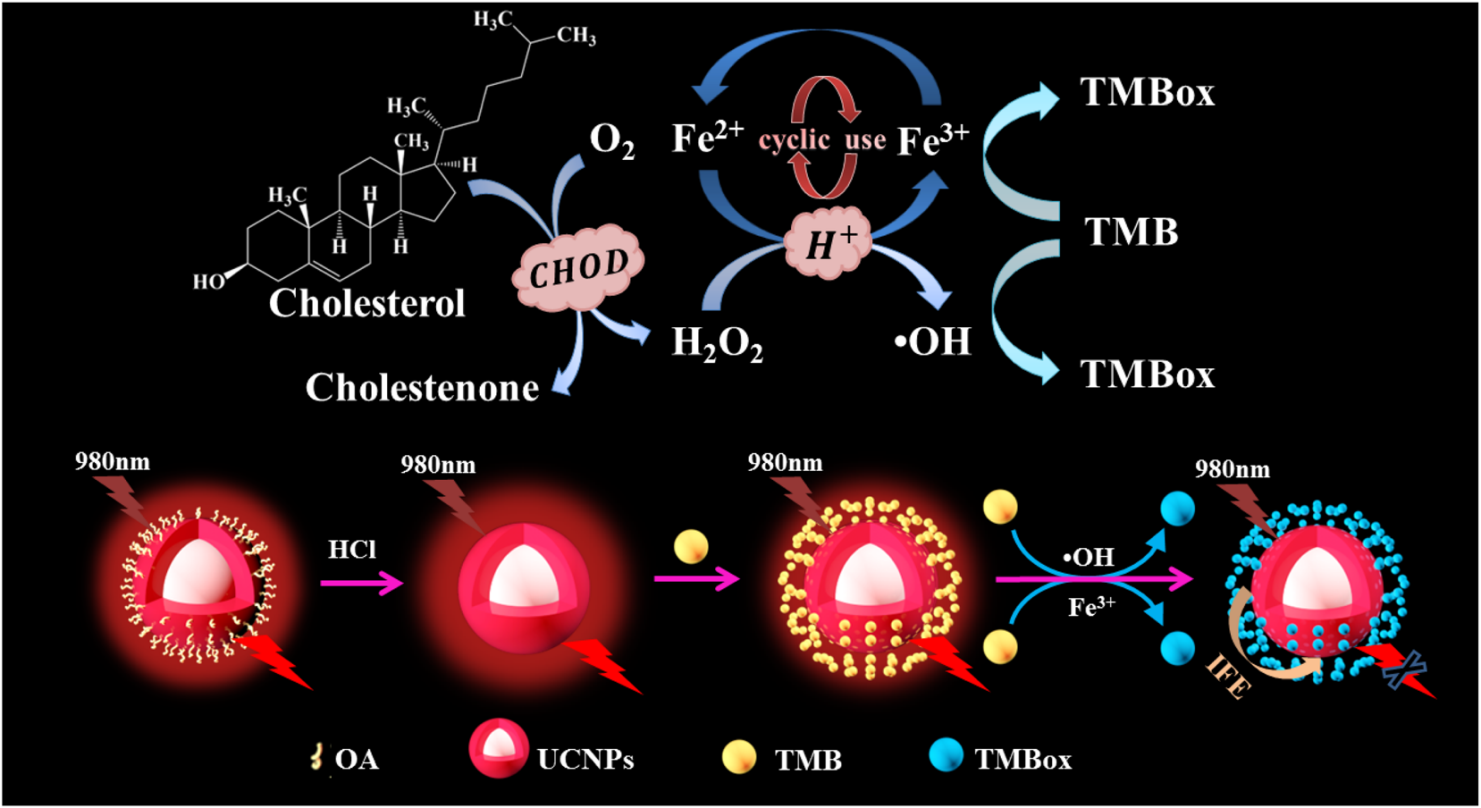
Detection mechanism diagram of CHOL.

Herein, a red UCNP-based sensor platform was developed for the detection of CHOL through coupling the cycle signal amplification strategy and the mechanism of detecting CHOL is shown in Scheme 1. First, CHOL is catalyzed by cholesterol oxidase (CHOD) to produce hydrogen peroxide (H_2_O_2_), which oxidizes Fe^2+^ to generate Fe^3+^ and the hydroxyl radical (·OH). Then 3,3′,5,5′-tetramethylbenzidine (TMB) is oxidized by the Fe^3+^ and ·OH produced to TMBox, during which Fe^2+^ is generated and can be recycled for producing TMBox. Finally, TMBox can effectively quench the red light emitted by UCNP through the internal filtration effect (IFE). The practicability and analysis principle of the developed assay for detecting CHOL were explored in detail and the effect of some detection condition on the sensing system was also studied. Finally, the proposed sensing platform was used to measure CHOL in actual serum samples, and the detection data obtained by colorimetry and fluorescence were compared.

## 2 Materials and methods

### 2.1 Materials

Gadolinium chloride hexahydrate (GdCl_3_·6H_2_O), ytterbium chloride (YbCl_3_·6H_2_O), thulium chloride hexahydrate (TmCl_3_·6H_2_O), Erbium chloride hexahydrate (ErCl_3_·6H_2_O), ammonium fluoride (NH_4_F), oleic acid (OA) and 1-octadecene (1-ODE) were obtained from Shanghai Aladdin Biochemical Technology Co., Ltd. (Shanghai, China) (Shanghai, China). 3,3′,5,5′-Tetramethylbenzidine (TMB), cholesterol (CHOL) and cholesterol oxidase (CHOD) were gotten from Aladdin Biochemical Reagent Co. Ltd. (Shanghai, China). Sodium hydroxide, hydrochloric acid, methyl alcohol, ethyl alcohol, cyclohexane, phosphate, FeCl_2_ and hydrogen peroxide (H_2_O_2_) were bought from Sinopharm Chemical Reagent Co. Ltd (Shanghai, China). Triton X-100 was purchased from Sigma-Aldrich (Shanghai) Trading Co., Ltd. Prepare a cholesterol stock solution by adding Triton X-100 to dissolve the cholesterol solid and dilute it with phosphate buffer saline (PBS) (10 mM, pH 7.4).

### 2.2 Measurements

The morphology of UCNP is tested by transmission electron microscopy using a Talos F200i apparatus (Carl Zeiss AG, Germany). UV-vis absorption and fluorescence spectrums of different samples were determined on a Lambda 950 UV spectrophotometer (PerkinElmer, USA) and a FluoroMax-4 spectrophotometer (Horiba Jobin Yvon, France) with laser excitation at 365 or 975 nm (Changchun New Industries, China), respectively. The crystalline phase of the prepared material was tested by an Ultima III X-ray diffractometer (Rigaku, Japan). Fourier transmission infrared spectra of UCNP were tested using a Nicolet-5700 infrared spectrometer (Thermo Elemental, USA).

### 2.3 Preparation of single red UCNP

The preparation of core–shell NaErF_4_ : 0.5% Tm@NaGdF_4_ : 40% Yb UCNP was referred to the reported literature.^17^ As UCNPs is used in the biological detection system in this work, water-soluble core–shell UCNP is obtained by removing OA from the surface of core–shell UCNP with hydrochloric acid. The detailed steps are as follows. The OA-UCNP dispersion is added to an equal amount of 2 M HCl solution and ultrasound is performed for 30 min. After centrifugation and washing, the ligand-free hydrophilic UCNP is obtained.

### 2.4 Fluorescent and colorimetric assay for the detection of H_2_O_2_

For H_2_O_2_ recognition by the fluorescent method, 20 μL of UCNP suspension (1 mg mL^−1^), 100 μL of HAc–NaAc buffer solution (100 mM, pH 3.6), 200 μL of H_2_O_2_ solution of different concentrations (0.5, 1.0, 5.0, 10, 50, 80 μM), 50 μL of 180 μM Fe^2+^, and 30 μL of TMB (4 mM) were blended. After reaction for 15 min and diluting to 500 μL with water, the luminous intensity was determined under 980 nm excitation.

For H_2_O_2_ recognition by the colorimetric method, 50 μL of UCNP suspension (1 mg mL^−1^), 200 μL of HAc–NaAc buffer solution (100 mM, pH 3.6), 400 μL of H_2_O_2_ solution with different concentrations (1, 5, 10, 35, 50, 80 μM), 100 μL of 180 μM Fe^2+^, and 60 μL of TMB (4 mM) were mixed. After incubation for 15 min and diluting to 2000 μL with water, the UV absorption intensity was determined.

### 2.5 Fluorescent and colorimetric assay for CHOL detection

The fluorescent assay for CHOL was realized under the following procedures. 200 μL of PBS (10 mM, pH 7.4) containing different concentrations of cholesterol (1, 15, 25, 60, 110, 160, 195 and 225 μM) was mixed with 50 μL of CHOD solution (1 mg mL^−1^). After reaction for 45 min, add 100 μL of HAc–NaAc buffer solution (100 mM, pH 3.6), 50 μL of Fe^2+^ (180 μM), 30 μL of TMB (4 mM) and 20 μL of UCNP (1 mg mL^−1^). After reaction for 15 min and diluting to 500 μL with water, the luminous intensity was determined under 980 nm excitation.

The colorimetric assay for CHOL was realized under the following procedures. 400 μL of PBS (10 mM, pH 7.4) containing different concentrations of cholesterol (1, 3, 5, 10, 20, 25, 55, 115, 175 and 220 μM), and 100 μL of CHOD solution (1 mg mL^−1^) were blended and reacted for 45 min. Then add 200 μL of HAc–NaAc buffer solution (100 mM, pH 3.6), 100 μL of Fe^2+^ (180 μM), 60 μL of TMB (4 mM) and 40 μL of UCNP (1 mg mL^−1^). After incubation for 15 min and diluting to 2000 μL with water, the intensity of UV absorption was determined.

### 2.6 Detection of CHOL in actual samples

At room temperature, the blood coagulated naturally for 10–20 min, centrifuged at 10 000 rpm for 10 min, collected the supernatant and diluted 100 times with phosphate buffer solution (10 mM, pH 6.7). The follow-up procedure for the detection of CHOL levels in serum is the same as in Section 2.5.

## 3 Results and discussions

### 3.1 Characteristics of UCNP

**Fig. 1:**
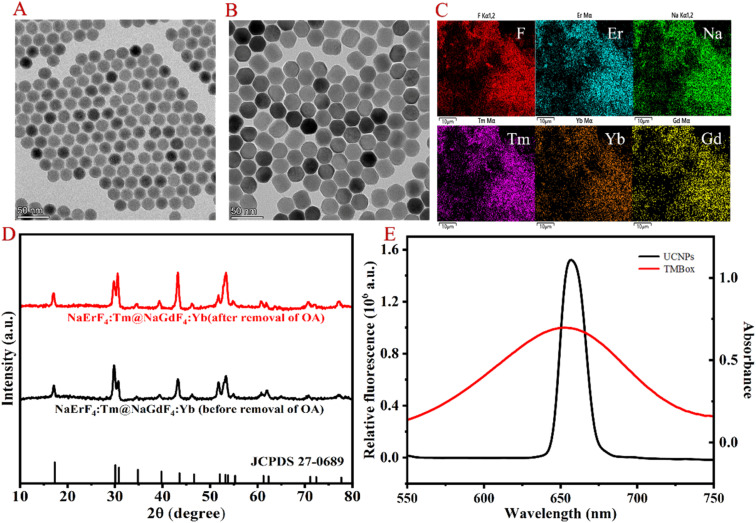
Morphological characterization and spectrum of UCNP. TEM (A and B), EDS (C), XRD (D) and fluorescence spectrum (E) of the prepared UCNP.

The morphological structure of the resultant UCNP was observed by transmission electron microscopy (TEM), energy dispersive spectrometer (EDS), X-ray diffractometry (XRD) and spectral properties. As shown in Fig. 1A and B, the TEM images clearly show that the prepared UCNP nanocrystals are uniformly dispersed. EDS analysis (Fig. 1C) indicates that the nanoparticles are mainly composed of Na, F, Er, Tm, Yb and Gd. Compared to the XRD pattern of the NaErF_4_ standard card, it can be seen in Fig. 1D that the crystal structure of UCNP is the same as that of NaErF_4_, which is a standard pure hexagonal phase with high crystallinity. As shown in Fig. 1E, the prepared UCNP emits strong red light at 654 nm under 980 nm excitation (black line), which can be attributed to the ^4^F_9/2_–^4^I_15/2_ transitions of Er^3+^.^18^ While TMBox has a wide UV-vis absorption spectrum at 550–750 nm (red line), which overlaps well with the red light emitted by UCNP and can effectively quench the fluorescence of UCNP. Therefore, a sensing platform for detecting CHOL is constructed using TMBox as the energy receptor and UCNP as the energy donor.

### 3.2 Feasibility and detection mechanism

**Fig. 2:**
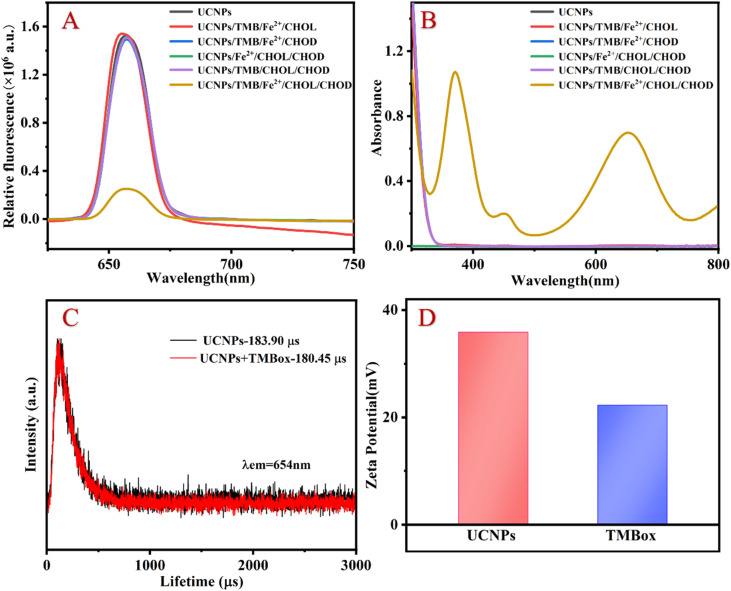
Fluorescent (A) and UV-vis spectra (B) of the different samples, the fluorescent lifetime of UCNP before and after TMBox (C) and the zeta potential of UCNP and TMBox (D). Experimental conditions: [UCNP]: 1 mg mL^−1^; [Fe^2+^]: 250 μM; [TMB]: 5 mM; [H_2_O_2_]: 200 μM; [Fe^3+^]: 250 μM; [HAc–NaAc buffer]: 100 mM, pH 3.6.

To explore the feasibility of the proposed method, the fluorescent and UV-vis spectroscopy of the different samples were performed. When CHOL exists, it reacts with CHOD to generate H_2_O_2_, converting Fe^2+^ to Fe^3+^ and ·OH. TMB is oxidized to TMBox by Fe^3+^ and ·OH. The generated TMBox can effectively quench the fluorescence of UCNP, resulting in a significant quenching of the 654 nm fluorescence emission peak and a corresponding increase in the UV absorption peak around 654 nm (as shown in Fig. 2A and B). Therefore, the proposed assay for detecting CHOL through a fluorescent and colorimetric dual signal is feasible.

The quenching mechanism may be attributed to fluorescence resonance energy transfer (FRET) or IFE.^19^ The difference between FRET and IFE is manifested mainly in two aspects: (1) the fluorescence lifetime in FRET decreases, while the fluorescence lifetime in IFE does not change^20^ and (2) the distance between the energy donors and receptors in FRET should be short enough (usually less than 10 nm) to allow the energy transfer process to occur, while the distance between the two materials in IFE is not relevant.^21^ To investigate the luminescence quenching mechanism of UCNP by TMBox, the luminescence lifetime of UCNP was investigated with and without TMBox, respectively. Fig. 2C shows that the fluorescence lifetime of a single UCNP solution is approximately 183.90 μs and the fluorescent lifetime of the mixed solution of UCNP and oxTMB is approximately 180.45 μs, which is almost the same. Therefore, IFE is the quenching mechanism of TMBox on UCNP. To further confirm this mechanism, the distance between energy donors and receptors can be explained by measuring the surface charges of UCNP and TMBox using the zeta potential. Fig. 2D shows that the zeta potentials of UCNP and TMBox are +35.9 mV and +22.2 mV, respectively. Due to electrostatic repulsion, the space between TMBox and UCNP could be greater than 10 nm,^22^ which is not conducive to the occurrence of FRET.

### 3.3 Optimization of the determination conditions

**Fig. 3:**
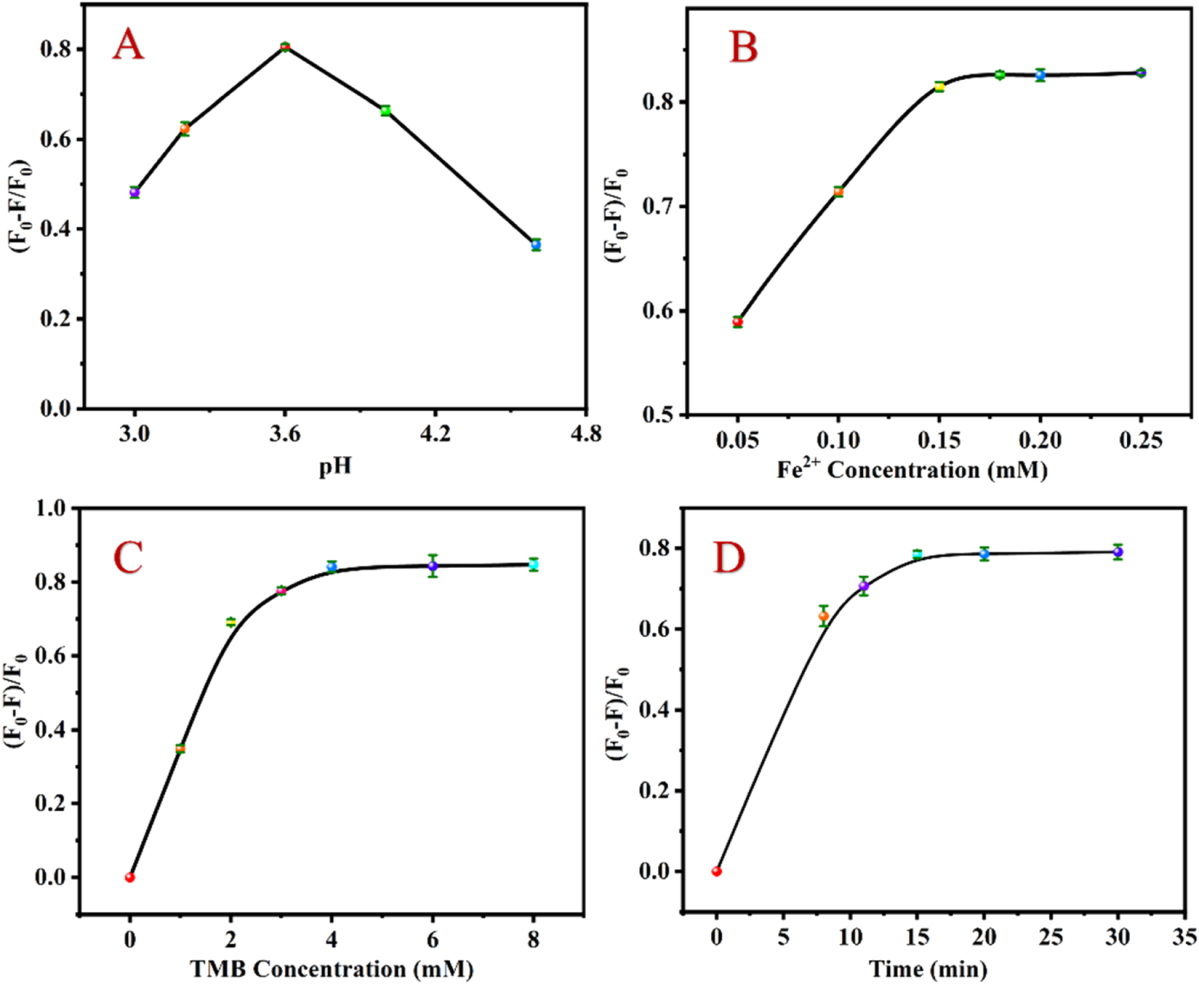
Effects of pH (A), Fe^2+^ concentration (B), TMB concentration (C) and reaction time (D) on ((*F*_0_ − *F*)/*F*_0_) of the detection system.

According to the analysis mechanism described above, when CHOL exists, it reacts with CHOD to generate H_2_O_2_, converting Fe^2+^ to Fe^3+^ and ·OH. Under acidic conditions, Fe^3+^ and ·OH by the Fenton reaction can oxidize TMB to TMBox, effectively quenching the luminescence of UCNP. To achieve the best detection effect, the relevant detection conditions were studied. Under the following experimental conditions, the relative luminescence quenching rate ((*F*_0_ − *F*)/*F*_0_) of the sensing system (*F*_0_ and *F* express the fluorescent intensity values before and after CHOL addition, respectively) attains the optimal value: pH value is 3.6 (Fig. 3A), Fe^2+^ concentration is 0.18 mM (Fig. 3B); TMB concentration is 4 mM (Fig. 3C); the reaction time is 15 min (Fig. 3D).

### 3.4 Detection of H_2_O_2_

**Fig. 4:**
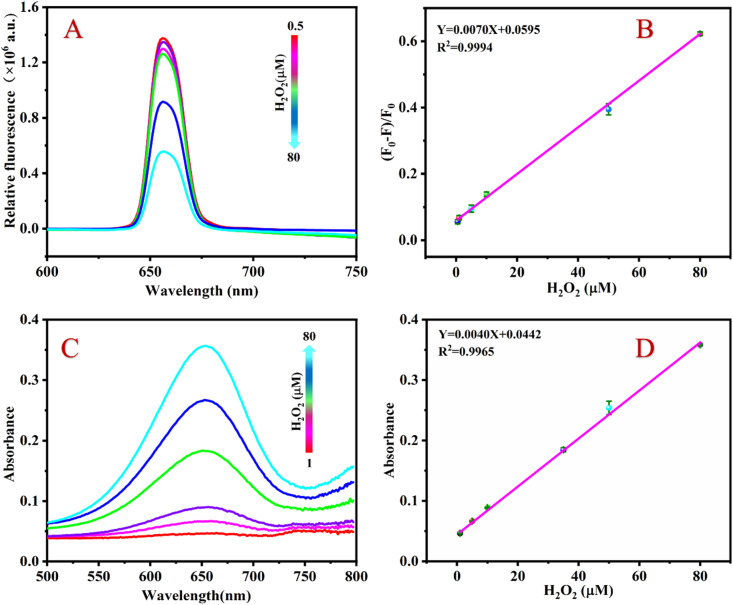
Fluorescence spectra of the detection system with increasing concentration of H_2_O_2_ (A), relationship between the (*F*_0_ − *F*)/*F*_0_ value and the concentration of H_2_O_2_ (B), absorbance spectra of the detection system with increasing concentration of H_2_O_2_ (C) and relationship between relative absorption intensity and concentration of H_2_O_2_ (D). Experimental conditions: [UCNPs]: 1 mg mL^−1^; [Fe^2+^]: 180 μM; [TMB]: 4 mM; [HAc–NaAc buffer]: 100 mM, pH 3.6.

Fig. 4A shows that the fluorescence intensity of the detection system continuously decreases at 654 nm when the H_2_O_2_ concentration gradually increases. As shown in Fig. 4B, if the H_2_O_2_ concentration increases from 0.5 to 80 μM, the value of (*F*_0_ − *F*)/*F*_0_ of the detection system continuously increases, furthermore, there is a good linear relationship between the value of (*F*_0_ − *F*)/*F*_0_ and the H_2_O_2_ concentration. The linear regression equation is *Y* = 0.0070*X* + 0.0595 with a correlation coefficient of 0.9994. According to the formula LOD = 3*σ*/*K* (where *s* is the slope of the standard curve, *σ* is the standard deviation), the detection limit (LOD) of H_2_O_2_ is calculated to be 0.33 μM.

Under optimal conditions, the sensing platform can also detect H_2_O_2_ by colorimetry. Fig. 4C shows that the absorbance value of the detection system at 654 nm gradually increases as the H_2_O_2_ concentration increases. Within the range of 1 to 80 μM, there is a good linear relationship between the H_2_O_2_ concentration and the absorbance value of the sensing platform. The linear equation is *Y* = 0.0040*X* + 0.0442 with a correlation coefficient of *R*^2^ = 0.9965 (Fig. 4D) and an LOD of 0.66 μM.

### 3.5 Detection of CHOL

**Fig. 5:**
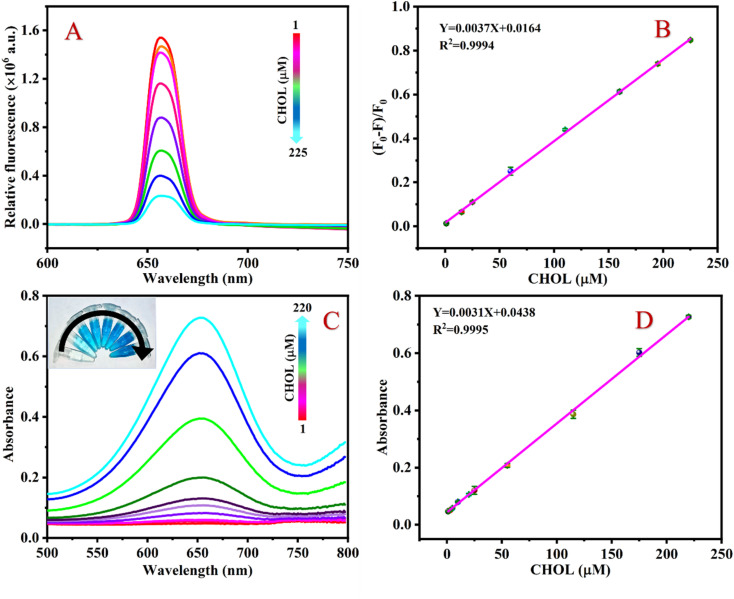
Fluorescence of the detection system with increasing concentration of CHOL (A), relationship between relative (*F*_0_ − *F*)/*F*_0_ value and concentration of CHOL (B), absorbance spectra of the detection system with increasing concentration of CHOL (C) and relationship between relative absorption intensity and concentration of CHOL (D). Experimental conditions: [UCNPs]: 1 mg mL^−1^; [Fe^2+^]: 180 μM; [TMB]: 4 mM; [HAc–NaAc buffer]: 100 mM, pH 3.6.

Under the best conditions, the different concentrations of CHOL were detected by the fluorescence-based sensing platform. Fig. 5A shows that the fluorescence intensity of the detection system continuously decreases at 654 nm as the concentration of CHOL gradually increases. As shown in Fig. 5B, as the concentration of CHOL gradually increased from 1 to 225 μM, the value of (*F*_0_ − *F*)/*F*_0_ of the detection system continues to increase and there is a good linear relationship between the value of (*F*_0_ − *F*)/*F*_0_ and the CHOL concentration, according to the linear regression equation *Y* = 0.0037*X* + 0.0164 with a correlation coefficient of 0.9994. By formula LOD = 3*σ*/*K*, the LOD of CHOL is calculated to be 0.63 μM.

Similarly, the response of the colorimetry-based sensing platform to different concentrations of CHOL was also measured. Fig. 5C shows that the absorbance value of the detection system at 654 nm gradually increases as the CHOL concentration increases. There is a good linear relationship between CHOL concentration in the range of 1–220 μM and the absorbance value of the detection system. The linear equation is *Y* = 0.0031*X* + 0.0438 with a correlation coefficient of 0.9998 (Fig. 5D) and the LOD is 0.85 μM. A comparison of the analytical performance of this method with previously reported sensors based on various nanomaterials was conducted to detect CHOL. The results show that compared to other CHOL sensors reported in the literature, the LOD of the sensing platform proposed in this work is lower or equivalent (Table 1).

**Table 1:** Comparison of reported strategies and this work detection for CHOL^a^

Material	Detection method	Linear range (μM)	Limit of detection (μM)	References
BNNS@CuS	Colorimetry	10–100	2.9	23
PB/MWCNT	Colorimetry	4–100	3.01	24
AuNCs	Fluorescence	10–100	5.77	25
Grp/b-CD/rhodamine 6G	Fluorescence	5–30	5	26
β-CD-CQD	Fluorescence	0–110	0.7	27
UCNPs	Fluorescence	1–225	0.63	This work
	Colorimetry	1–220	0.85	

a Boron nitride nanosheet and copper sulfide nanohybrids: BNNS@CuS, Prussian blue: PB, multi-walled carbon nanotubes: MWCNT.

### 3.6 Specific verification

**Fig. 6:**
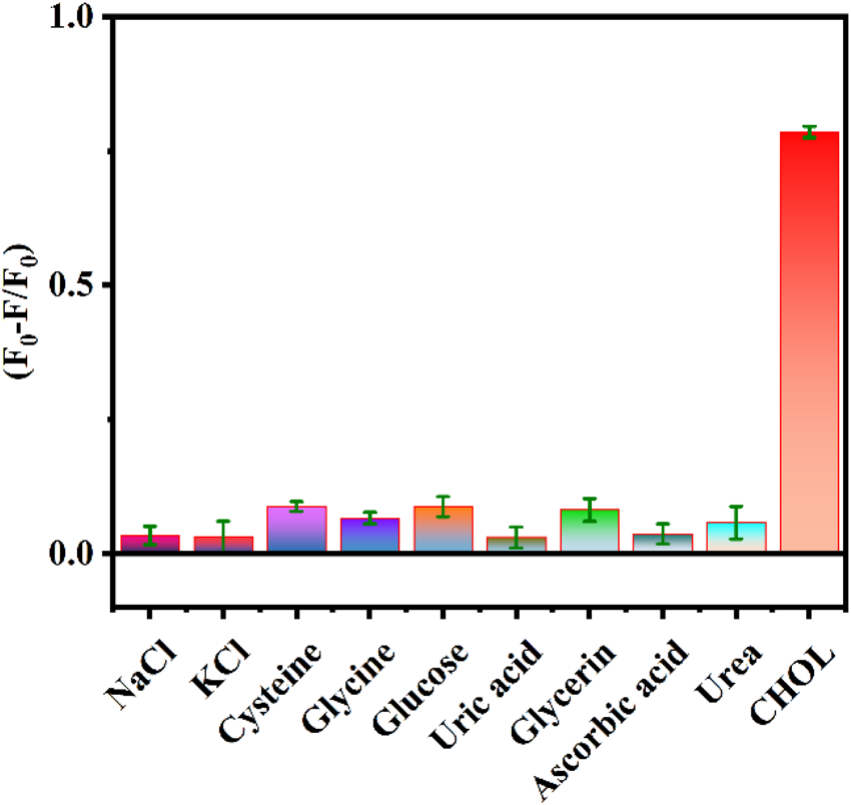
Effects of interfering substances on the (*F*_0_ − *F*)/*F*_0_ value of the detection system. Selectivity of the proposed sensing platform. Experimental conditions: [CHOL]: 200 μM, [NaCl]: 1 mM, [KCl]: 1 mM, [cysteine]: 200 μM, [glycine]: 1 mM, [glucose]: 1 mM, [uric acid]: 200 μM, [glycerin]: 1 mM, [ascorbic acid]: 200 μM, [urea]: 1 mM.

To verify the specificity of the sensing platform for CHOL detection, other possible interfering substances in the serum such as NaCl, KCl, cysteine, glycine, glucose, uric acid, glycerin, ascorbic acid, and urea were analyzed as controls. Fig. 6 shows that there is no obvious change in the values of (*F*_0_ − *F*)/*F*_0_ in the presence of other interfering substances, while the values of (*F*_0_ − *F*)/*F*_0_ significantly increases in the presence of CHOL. The above results indicate that the proposed assay has good selectivity for detecting CHOL and is expected to be used for efficient detection of CHOL in actual samples.

### 3.7 Detection of CHOL in human serum

To evaluate the practicality of the proposed detection system, CHOL was detected in human serum samples. First, the actual concentration of CHOL was measured in diluted human serum samples. Table 2 shows that the measured CHOL values in the diluted serum samples of (19.90 and 35.41 μM, fluorescence method), (19.73 and 35.15 μM, colorimetric method). To further investigate the accuracy, stability, and actual detection performance of the sensing platform, the CHOL spiked recovery experiment was conducted. After adding different concentrations of standard CHOL samples (1, 50 and 100 μM) to human serum samples, the measured recoveries range from (97.2% to 102.2%, fluorescence method), (97.1% to 103.7%, colorimetric method) and the relative standard deviation (RSD) (*n* = 3) range from (1.23% to 3.21%, fluorescence method) (1.22% to 3.76%, colorimetric method). These results indicate that the proposed sensing platform can be used for the analysis of CHOL in actual biological samples and provide the possibility for real-time and accurate detection of CHOL in actual biological samples.

**Table 2:** Detection results of CHOL in human serum samples

Samples	Added (μM)	Fluorescence			Colorimetry		
		Found (μM)	Recovery (%)	RSD (%)	Found (μM)	Recovery (%)	RSD (%)
Serum 1	0	19.90	—	1.59	19.73	—	2.10
	1	20.88	97.4	3.21	20.73	99.9	1.50
	50	70.91	102.0	1.23	71.58	103.7	1.34
	100	119.40	98.5	2.65	121.40	101.7	2.21
Serum 2	0	35.41	—	2.25	35.15	—	1.22
	1	36.41	99.9	2.32	36.16	100.8	3.76
	50	84.03	97.2	1.93	84.69	97.1	2.37
	100	137.66	102.2	1.63	136.29	101.1	2.87

## 4 Conclusions

A dual mode signal-sensing platform based on fluorescence and colorimetry has been constructed for the quantitative detection of CHOL in biological samples using UCNP with strong single-infrared emission as the luminescent material and TMBox as the quenching agent. The practicability and analysis principle of the developed assay for detecting CHOL were explored in detail and the effect of some detection condition on the sensing system was also studied. Compared to traditional biosensors, the proposed sensing platform has a better response through the coupling IFE and cyclic amplification strategy. Under the best experimental conditions, the LOD of CHOL by the fluorescence and colorimetry-based sensing platform are 0.63 and 0.85 μM, respectively. The quantitative detection of CHOL in human serum shows that the developed assay has good accuracy and stability. The sensing platform constructed in this work is expected to offer new ideas for the development of CHOL clinical detection methods that are easy to operate, highly sensitive and selective.

## Ethical statement

Human serum samples were collected from Affiliated Cancer Hospital of Guangzhou Medical University. Before laboratory study, written informed consent was signed by all volunteers. All experiments were performed in accordance with the guidelines approved by Affiliated Cancer Hospital of Guangzhou Medical University. The research protocol (no. GYZL-ZN-2023(053)) was approved by Medical Ethics Committee of Affiliated Cancer Hospital of Guangzhou Medical University.

## Author contributions

Pang Tingyuan, Liu Xiaorui and Zhang Meimei designed research. Pang Tingyuan, Liu Xiaorui, Li Jia and Song Qi performed the experiments. Li Junren, Han Ling, Shu Wenying and Jian Xiaoshun analyzed data. All author wrote and revised the manuscript.

## Conflicts of interest

The authors declare that they have no known competing financial interests or personal relationships that could have appeared to influence the work reported in this paper.

## Supplementary Material

RA-014-D3RA07354H-s001
