# Supplementary material for: Highly sensitive and accurate detection of cholesterol based on a single red upconversion biosensor
Source: RSC Adv. 2024 Mar 6;14(11):7858–66. doi: 10.1039/d3ra07354h (PMC10915588; doi:10.1039/d3ra07354h)
Supplement: RA-014-D3RA07354H-s001 [file RA-014-D3RA07354H-s001.pdf]

Electronic Supplementary Material (ESI) for RSC Advances.
This journal is © The Royal Society of Chemistry 2024

Supporting Information

Highly sensitive and accurate detection of cholesterol based on single red upconversion biosensor

Pang Tingyuan[1], Liu Xiaorui[1], Li Jia[1], Song qi[1], Li Junren[1], Han Ling[2], Shu Wenying[1], Jian Xiaoshun[1], Zhang Meimei[1]*

[1]Department of Pharmacy, Affiliated Cancer Hospital & Institute of Guangzhou Medical University, Guangzhou, China

[2]Integrated Traditional Chinese and Western Medicine Department (Internal Medicine Section 5), Affiliated Cancer Hospital & Institute of Guangzhou Medical University, Guangzhou, China

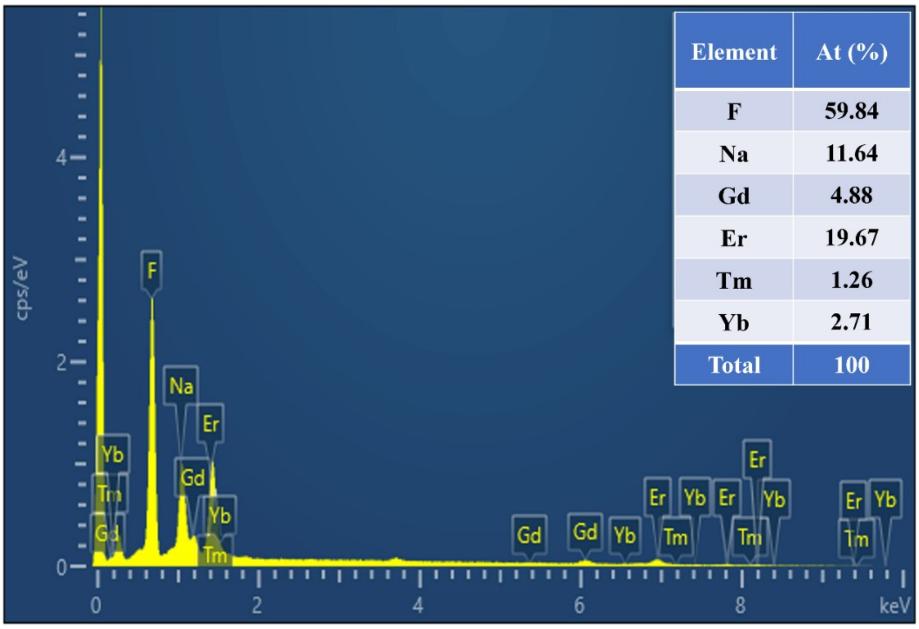

Fig. S1 Element distribution of $NaErF_4$:0.5%Tm@$NaGdF_4$:40%Yb UCNP

The infrared spectrums of the prepared nanomaterials (**Fig. S2)** show the -OH stretching vibration peak of the OA molecule corresponding to 3432 cm^{-1}, the -CH_2 stretching vibration peak of the OA molecule corresponding to 2927 and 2858 cm^{-1}, and the -COO stretching vibration peak corresponding to 1564 and 1463 cm^{-1} become weak or even disappear. These indicate that most of the OA molecules attached to the surface of UCNPs have been removed.

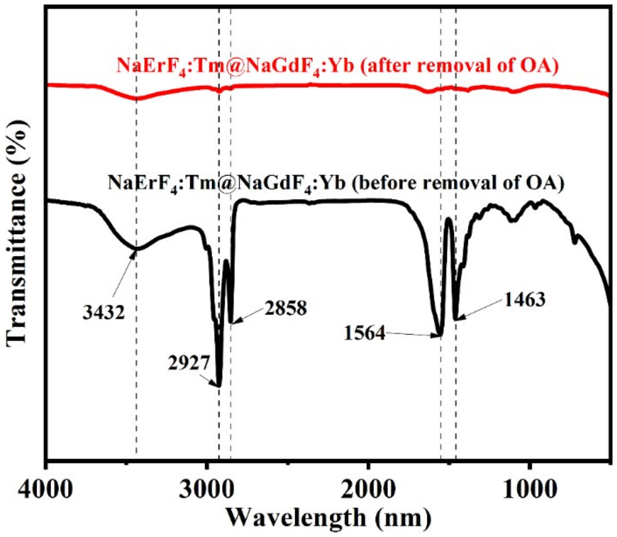

Fig. S2 The infrared spectrums of the prepared UCNPs
